# Dietary supplementation of germinated pigmented rice (*Oryza sativa* L.) lowers dyslipidemia risk in ovariectomized Sprague–Dawley rats

**DOI:** 10.3402/fnr.v60.30092

**Published:** 2016-03-30

**Authors:** Lara Marie Pangan Lo, Mi Young Kang, Seong Joon Yi, Soo Im Chung

**Affiliations:** 1 Department of Food Science and Nutrition, Brain Korea 21 Plus, Kyungpook National University, Daegu, Republic of Korea; 2 College of Veterinary Medicine, Kyungpook National University, Daegu, Republic of Korea

**Keywords:** germinated rice, lipid metabolism, pigmented rice, ovariectomy, Sprague–Dawley rats

## Abstract

**Background:**

In the recent years, cases of elderly women suffering from metabolic diseases such as dyslipidemias brought about by hormonal imbalance after menopause are continuously increasing. In this regard, a continuous and escalating demand to develop a more functional and highly nutritional food product as an adjunct supplement that can help alleviate these diseases is still being sought.

**Objective:**

This study investigated the effects of germinated blackish-purple rice cultivars *Keunnunjami*, *Superjami*, and reddish-brown cultivar *Superhongmi* in the lipid metabolism of ovariectomized Sprague–Dawley rats.

**Method:**

The animals were randomly divided into nine groups (*n*=5) and were supplemented with either non-germinated or germinated rice for 9 weeks. Then the plasma, liver, and fat samples were collected for the lipid metabolism effects analyses.

**Results:**

Animals fed with germinated rice cultivars had improved lipid profile levels relative to the groups supplemented with non-germinated rice cultivars. The germinated rice groups, *Keununjami* and *Superjami* in particular, showed a low total cholesterol levels, high levels of high-density lipoproteins-cholesterol, high fecal lipid output, low hepatic lipid values, and low hepatic adipocyte accumulation. There was also an increase in the rate of lipolysis and decrease in lipogenesis based on the lipid-regulating enzyme activity profiles obtained for the groups that fed on germinated rice. Also, results revealed that pigmented rice cultivars had superior effects in improving the lipid metabolism relative to the non-pigmented normal brown rice variety.

**Conclusion:**

Based on the results, this study suggests that germinated pigmented rice consumption can confer better lipid metabolism than ordinary white rice and constitutes as an effective functional food in alleviating the risk of having dyslipidemias like those suffering from menopausal co-morbidities.

Dyslipidemia is defined as abnormal high levels of dietary lipids such as plasma cholesterol, triglycerides, or a low level of high-density lipoproteins (HDL). In addition, dyslipidemia often increases the risk factor for atherosclerosis, which further increases the risk for developing coronary heart diseases that are the leading cause of death among the aged population (1). As people age, their risk for developing metabolic diseases also increases. In fact, according to the National Health and Nutrition Examination Survey (NHANES) 2003–2006, 52.9% of adults in the United States had lipid abnormalities and the number is expected to increase in the next few years (2). Also, in China alone, elderly people have higher prevalence of hyperlipidemia, with total cholesterol (TC) levels greater than 240 mg/dL in approximately 25% of men and 42% of women (3). Elderly women, mainly those who are on their menopausal stage, suffer a greater risk of having lipid abnormalities due to decreasing levels of estrogen. Guetta and Cannon III (4) elucidated that, before menopause, plasma cholesterol levels are lower and HDL-cholesterol levels are higher in women compared with men of the same age group. They also added that, after experiencing menopause, women's plasma cholesterol levels increase abruptly, commonly exceed those of same-aged men, and have smaller, denser, and potentially more atherogenic particle sizes, thus increasing atherogenic risks further. Also, their plasma HDL-cholesterol levels decline after menopause (4). Although hormone replacement therapy has been widely available and is accepted as the gold standard for estrogen replacement, alternative and preventive treatments that are less costly but more effective are continuously being sought.

Functional foods, such as whole grains, have been developed in recent years to help alleviate and reduce the risk of many metabolic diseases. Rice is the most cultivated grain almost in all areas of the world. It is also one of the staple foods in every household in Asia. Furthermore, rice has gained a lot of interest in the recent years because it has high nutrient and mineral contents as well as bioactive compounds. Thus, studies have become more focused on the development and breeding of different rice varieties to enhance its nutrients (5).

Pigmented rice varieties are known to contain higher number of anthocyanins, γ-oryzanol, ferulic acids, and unsaponifiable compounds compared to non-pigmented rice cultivars. These compounds are believed to lower the cholesterol and triglycerides levels (6). Also, pigmented rice varieties are believed to have higher bioactive compounds, such as γ-oryzanol and tocopherols, that can help in the upregulation of cholesterol synthesis and catabolism activities (7). On the other hand, Juliano (8) defined germination as a process of the emergence of an embryonic plant from a seed. When rice grains are germinated, they experience structural changes including the degradation of starch and protein reserves of the endosperm and an increase in enzyme activities, which result in biochemical changes such as increased nutrient and mineral content, as well as enhanced bioactive compounds (8).

Consumption of pigmented rice is believed to have more nutritional benefits than the normal white rice in lowering cholesterol levels. Similarly, subjecting rice to the germination process will increase these nutritional benefits even more. However, there are limited data on the effects of germinated rice grains on various metabolic diseases like dyslipidemia and other conditions brought about by menopause. Combining these two parameters, the newly developed Korean purple rice varieties *Keunnunjami* (KJ) and *Superjami* (SJ) and red rice *Superhongmi* (SH) were germinated for 72 h and their effects on lipid metabolism were therefore studied on this research.

## Materials and methods

### Standards, kits, and chemicals

All other standards and chemicals used in this study were of analytical and HPLC grade and were purchased from the Sigma Chemical, Co. (St. Louis, MO, USA) or Merck KGaA (Darmstadt, Germany).

### Rice samples

Newly bred purple rice cultivar *Keunnunjami* (*KJ*) and *Superjami* (*SJ*), and red rice cultivar *Superhongmi* (*SH*) were used in this study and compared to non-pigmented *Normal Brown Rice* (*NB*). These rice cultivars were all obtained from the Department of Agricultural Science, Korea National Open University (Seoul, Korea). They were grown between May and October 2014 at Jochiwon-eup, Sejong City, Korea.

### Non-germinated rice samples

Two hundred and fifty grams of rice samples were weighed and washed twice with distilled water thoroughly to remove any dirt. The rice grains were placed in an oven tray topped with paper towel and placed in an oven at 50°C for 2 h to lower the moisture content (WiseVen^®^, PMI-Labortechnik GmbH, Deutschland) at an approximate of 13–15%. The dried rice grains were grinded, pulverized (HMF-3250S, Hanil Electronics, Seoul, Korea), passed through 100-mesh sieve and packed in hermetically sealed Ziploc^®^ plastic bags (Bangkok, Thailand), and stored at −20°C until further treatment.

### Germinated rice samples

Germination was done following the method of Wu et al. (9) with minor modifications. Briefly, 250 g of each of the rice samples was weighed and washed two times thoroughly with distilled water. After washing, the rice grains were placed evenly in a tray overlaid with cotton pads and cheesecloth to maintain a damp condition. Thereafter, enough water was added to completely soak the rice grains, and the whole tray was covered with a clean, transparent plastic wrap with holes to accommodate proper moisture and air circulation condition and was incubated at 30°C. The rice samples were checked every 12 h to ensure that no foul odor and fungal growth developed. After 72 h, the germinated rice grains were collected and dried in an oven (WiseVen^®^, PMI-Labortechnik GmbH, Deutschland, Germany) at 50°C for 2 h to lower the moisture content to 13–15%. The dried rice grains were grinded, pulverized (HMF-3250S, Hanil Electronics, Seoul, Korea), passed through 100-mesh sieve and packed in hermetically sealed Ziploc^®^ plastic bags (Bangkok, Thailand), and stored at −20°C until further treatment.

### Bioactive compounds

#### γ-Oryzanol

γ-Oryzanol in the rice powder samples was extracted in the rice samples and determined as described by Islam and Becerra (10). Briefly, 1.00 g of pulverized rice samples was extracted with 3.0 mL of HPLC-grade methanol. The mixture was shaken using a rotator mixer (Chang Shin Scientific, Co., Pusan, Korea) for 2 h. After the extraction, the samples were centrifuged at 825 rpm, 4°C for 10 min. The extraction process was repeated three times and the supernatants were then collected and filtered through 0.45-µM microfilter (Chromdisc^®^). Fifty microliters of the extracts were injected into Agilent HPLC (1200 Series, Japan) using C18 column (4.6×250 mm, 5 µm). The HPLC was equipped with a UV–Vis photodiode array detector set at 330 nm wavelength. Methanol/acetonitrile/dichloromethane/acetic Acid (50:44:3:3) were used as the mobile phase at room temperature. The flow rate was set at 1 mL/min. γ-Oryzanol standard was used for to calibrate and calculate the concentration in the rice samples.

#### Ferulic acid

Determination of ferulic acid was performed using the method described previously by Banchuen et al. (11). In summary, 0.5 g of pulverized rice sample was extracted using 10.0 mL of 1 M NaOH for 3 h at 40°C. The extract was then neutralized with 5.0 mL of 2 M HCl. Each of the samples was further extracted with 10.0 mL ethyl acetate for another 5 min. Extraction procedure was repeated three times. The ethyl acetate layer was collected and evaporated. Then, the extract was redissolved in methanol/water (1:1) mixture. After which, the extracts were filtered in 0.45 µM pore size syringe-driven filter (Chromdisc) before reversed-phase HPLC analysis. A 5.0-µL aliquot of the extract solution was separated using an Agilent HPLC system equipped with a photodiode array detector on a 4.6×150 mm, 5 µm, and Agilent C18 analytical column. The temperature of the column was set at 40°C and the wavelength used was 320 nm. The mobile phases used were 2.5% acetic acid and acetontrile (88:12) at a flow rate of 0.5 mL/min. Pure ferulic acid standard was used for calibration.

#### Tannins

Colorimetric estimation of tannin content was based on the blue color formed by the reduction of phosphotungstomolybdic acid by the tannin-like compound in alkaline medium and was analyzed using the method of Padma et al. (12). Basically, 200 µL of the methanolic extract was mixed with 1.5 mL of distilled water. Then, 100 µL of Folin–Denis Reagent and 200 µL of 0.5% Na_2_Co_3_ were added. The absorbance of the resulting solution was measured spectrophotometrically at 700 nm. The total tannic acid content was expressed as milligrams of tannic acid equivalent per 100 g of sample extract.

### In vivo lipid metabolism experiments

#### Experimental animals and diets

**Fig. 1:**
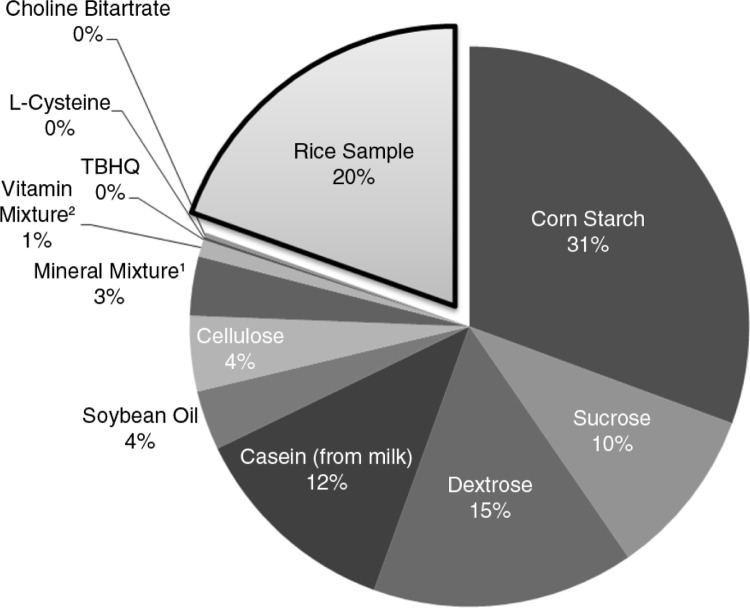
Composition of the experimental animal diet (based on AIN 93M semi-synthetic animal diet). ^1^AIN-93M mineral mixture (S10022M) – (based on 35 g/1,000 g diet): 5.0 g Ca, 2.0 g Pi, 0.5 g Mg, 3.6 g K, 0.3 g S, 1.0 g Na, 1.6 g Cl, 6.0 mg Cu, 0.2 mg I, 45.0 mg Fe, 10.5 mg Mn, 0.2 mg Se, and 30.0 mg Zn. ^2^AIN-93M vitamin mixture (V10037) – (based on 10 g/1,000 g diet): 4000 IU vitamin A palmitate, 1000 IU cholecalciferol, 75 IU vitamin E acetate, 0.75 mg phylloquinone, 0.2 mg biotin, 25 µg cyanocobalamin, 2 mg folic acid, 30 mg nicotinic acid, 16 mg calcium pantothenate, 7 mg pyridoxine-HCl, 6 mg riboflavin, and 6 mg thiamine HCl.

Forty-five female ovariectomized (OVX) Sprague–Dawley rats initially weighing an average of 228 g were purchased from Central Lab. Animal, Inc. (Seoul, Korea). They were housed in stainless-steel, ventilated cages in an animal room maintained at 25±2°C, relative humidity of 50±2%, and a 12-h light–dark cycle. After 1 week acclimatization, the OVX rats were randomly divided into nine diet groups (*n*=5). One group was fed with pelletized AIN-93M diet and served as the normal control (NC) group. Meanwhile, the treatment groups were fed with modified AIN-93M diet supplemented with 200.0 g of non-germinated and germinated NB, KJ, SJ, and SH rice/kg diet (Fig. 1), which corresponds to the daily dietary grain intake needs of menopausal women. The pelletized diet was given for 9 weeks and the rats were allowed to eat and drink distilled water *ad libitum*. Feed intake (Table 2) was measured daily while body weight was measured biweekly. At the end of the experimental period, all rats were deprived of food for 12 h and sacrificed by allowing each to sniff ethyl ether in a confined cage until immobilized. This animal experiment protocol was approved by the ethics committee for animal studies of Kyungpook National University (approval no. 2015-0087).

#### Internal body organs collection

The major organs like heart, liver, kidneys, and fat (inguinal and perirenal) were harvested, washed with physiological saline (pH 7.4), weighed, and stored immediately at −70°C until further analyses.

#### Blood biochemical analyses

The plasma TC, total triglycerides (TG), and HDL-cholesterol were analyzed according to the methods provided in the manufacturer's kit (Asan Pharmaceuticals, Seoul, Korea). The plasma-free fatty acids (FFA) and phospholipids were measured following the instructions of commercial kits (Enzychrom, BioAssay Systems, California, USA). Similarly, liver enzymes *glutamic oxaloacetate transaminase* (GOT) and glutamic pyruvate transaminase (GPT) levels were measured using the method of commercial kits (Asan Pharmaceuticals). Non-HDL cholesterol, HDL-cholesterol/triglyceride ratio (HTR) and atherogenic index (AI) were determined using the following formulas:

- Non-HDL cholesterol=TC - HDL cholesterol
- % HTR=(HDL cholesterol/TC)*100
- AI=(TC - HDL cholesterol)/HDL

#### Hepatic TC and TG assays

The extraction of hepatic lipids was based on the method by Seo et al. (13) with modifications. Briefly, 100 g of liver samples was analyzed using 30% potassium hydroxide and digested in boiling water bath for 30 min. After cooling, the resulting lysates were mixed with H_2_O/95% ethanol (1:1) and centrifuged for 10 min at 3,000 rpm at 4°C. The supernatant was collected and mixed with 1 M magnesium chloride. The whole mixture was incubated in ice and centrifuged again for 5 min. The resulting final supernatant was used to measure TC and TG using the experimental kit (Asan Pharmaceuticals) methods similar to plasma samples. The results were expressed as mg/g of liver sample.

#### Fecal TC and TG assays

The extraction of fecal lipid measurements was based on the method of Folch et al. (14) with modifications. Briefly, 0.5 g of powderized feces was extracted with 5.0 mL of chloroform/methanol (2:1) for 30 h at 4°C. Then, the mixture was centrifuged at 1,000 rpm for 15 min at 4°C. Using a stream of nitrogen gas, 2.5 mL of the supernatant was completely evaporated and redissolved using 1.0 mL of chloroform/methanol (2:1) solution. Then, 100.0 µL was completely dried again using nitrogen gas and redissolved with 5.0 mL of ethanol. A 50.0 µL of the extract was placed in a 2.0 mL eppendorf tube and 650.0 µL of emulsifier (0.5% Triton X-100 and sodium cholate mixture) was added. The resulting solution was used to measure TC and TG using the experimental kit (Asan Pharmaceuticals) methods similar to plasma samples. The results were expressed as mg/g of fecal sample.

#### Liver enzyme extraction

The liver and adipose tissue enzymes were extracted using the method of Hulcher and Olson (15). In summary, the organ tissues were homogenized using 5.0 mL of enzyme buffer composed of 0.1 M triethanolamine, 0.002 M dithiothreitol and 0.02 M ethylenediaminetetraacetic acid. The mixture was centrifuged (Beckman Coulter Korea Ltd, Seoul, Korea) at 3,000 rpm for 15 min at 4°C. The supernatant was collected and further centrifuged at 13,000 rpm for 20 min at 4°C. Meanwhile, the precipitate was resuspended with a 3.0-mL of enzyme buffer and centrifuged at 13,000 rpm for 20 min at 4°C. The resulting precipitate was redissolved using a 1.0-mL enzyme buffer for carnitoyl transferase (CPT) and β-oxidation (β-Ox) activity assays. Meanwhile, the supernatant was collected and further centrifuged at 32,500 rpm for 60 min at 4°C. The supernatant was collected for glucose-6-phosphate dehydrogenase (G6PD) activity, and the precipitate was resuspended using the same buffer for the analysis of fatty acid synthase (FAS) and malic acid dehydrogenase (ME) activities.

#### Lipid-regulating enzyme and β-oxidation activities

The CPT activities were measured using the mixture described by Bieber et al. (16). The reaction was initiated by the addition of a tissue sample and incubated at 25°C for 2 min. The change in absorbance at 412 nm was determined, and the activity was expressed as nmol oxidized CoA/min/mg protein. The G6PD activities of the samples were analyzed following the method of Pitkänen et al. (17) that was based on the reduction of 6.0 mM nicotinamide adenine dinucleotide phosphate (NADP^+^) by G6PD in the presence of glucose-6-phosphate. The changes in the absorbance of the samples at 340 nm at 37°C were determined. Moreover, the FAS activities were determined spectrophotometrically using Gibson and Hubbard's method (18). In brief, the assay mixture was reacted with the tissue sample and the change in absorbance measured at 340 nm at 30°C was recorded. Meanwhile, the ME activities were based on the method developed by Ochoa (19), wherein the reaction mixture was reacted with the tissue sample and the change in absorbance at 340 nm at 27°C was measured. The results of G6PD, FAS, and ME were expressed as nmol reduced NADPH/min/mg protein. Last, the β-Ox activities were measured from the final product of NADH by palmitoyl substrates based on the method of Lazarow (20). The reaction was initiated by the addition of 10 µL of tissue sample to the reaction mixture and incubated at 37°C for 5 min. The change in absorbance at 340 nm was measured and the activity was expressed as an nmol-reduced NADPH/min/mg protein.

#### Hepatic histochemistry

Hepatic histological examination followed the procedure of Posuwan et al. (21). The liver sample tissues were fixed in 10% neutral-buffered formalin. The tissue blocks were processed routinely, embedded in paraffin, and sectioned serially by 4–5 µm in thickness. Representative sections were stained with hematoxylin and eosin (H&E, Sigma–Aldrich, St. Louis, MO) for light microscopic examination at a magnification of 200×. Then the area of adipocytes (µm^2^) was obtained using Image J 1.48v software (NIH, Bethesda, MD) for each sample.

### Statistical analyses

All data were analyzed using one-way analysis of variance (ANOVA) by Statistical Package for Social Sciences software program version 22.0 for windows (SPSS, Inc., Chicago, IL). Values were reported as mean±standard deviation (SD). The difference between the means of all dietary groups was assessed using Tukey's multiple range test. The differences between the means of non-germinated versus germinated groups were evaluated using Student's independent *t*-test. Statistical significance was considered at *p<*0.05.

## Results

### Germination increases the bioactive components of rice

To determine the γ-oryzanol, ferulic acid, and tannins contents, germinated rice grains and non-germinated rice grains were subjected to HPLC and spectrophotometric technique, respectively. Among the rice cultivars, germinated pigmented KJ and SJ were shown to have the highest γ-oryzanol content among the four rice samples investigated (Table 1). The same trend was obtained for ferulic acid after the 3-day germination process. γ-Oryzanol is a mixture of ferulic acid esters of sterols and triterpene alcohols that is known to have hypolipidemic and antioxidant effects (6). Germination process had substantially increased the amounts of these bioactives. In contrast, the germinated reddish SH (3.65±0.03 mg TAE/100 g) had the highest tannin content among the rice cultivars investigated, followed by KJ (1.86±0.01 mg TAE/100 g), while tannin content was not detected in the NB variety. Overall, germinated pigmented rice had levels of bioactive compounds superior to those of the non-germinated and non-pigmented varieties.

**Table 1:** Nutritional composition of the germinated rice samples

		Non-germinated	Germinated
γ-Oryzanol (mg/g)	NB	0.21±0.01^a^	0.35±0.01^a^*
	KJ	0.28±0.02^b^	0.42±0.00^b^*
	SJ	0.37±0.00^d^	0.65±0.01^d^*
	SH	0.31±0.00^c^	0.52±0.01^c^*
Ferulic acid (mg/g)	NB	0.04±0.00^a^	1.15±0.01^a^*
	KJ	0.98±0.02^c^	2.25±0.02^d^*
	SJ	0.60±0.01^b^	1.84±0.01^c^*
	SH	0.61±0.00^b^	1.60±0.01^b^*
Tannins (mg TAE/g)	NB	ND	ND
	KJ	200.36±15.89^b^	1869.32±12.21^b^*
	SJ	156.26±10.36^a^	1652.47±25.87^a^*
	SH	1374.57±18.36^c^	3702.65±22.65^c^*

NB, normal brown rice; KJ, Keunnunjami; SJ, Superjami; SH, Superhongmi; ND, not detected; TAE, tannic acid equivalent.

Values are means±SD (*n*=5).

a–d Means in the same column per parameter with different letters are significantly different at *P*<0.05 by Tukey's range test.

* on the germinated rice group indicates significant difference (*P*<0.05) by independent *t*-test between germinated and non-germinated samples.

### Germinated rice consumption helps decrease total weight gain and adipose tissue weight

Table 2 shows the body weight gain, feed intake, FER, and organ weights of OVX rats. No significant difference was found in the initial weight of the rats after consuming non-germinated rice for 9 weeks. On the contrary, germinated rice consumption, especially those that fed on purple rice gain cultivars KJ and SJ, did not have excessive weight gain relative to the other groups. As for the organ weights, no significant difference was found between the kidney and liver weights of the animals that consumed non-germinated or germinated rice. However, the liver weights of the OVX rats in the germinated KJ and SH group were significantly lighter than the rats that consumed non-germinated rice. In addition, the supplementation of germinated rice in all groups lowered total fat pad weights compared to those of the rats that consumed non-germinated rice.

**Table 2:** Body weight gain and weights of organs and adipose of OVX rats fed with non-germinated and germinated rice grains

		Non-germinated	Germinated			Non-germinated	Germinated
Initial weight (g)	NC	231.33±10.12	231.33±10.12	Liver (g)	NC	12.47±0.87^b^	12.47±0.87^b^
	NB	229.00±9.64	227.33±8.62		NB	10.59±0.39^a^	10.64±0.82^ab^
	KJ	229.00±9.85	227.33±6.43		KJ	10.43±1.74^a^	8.91±0.78^a^*
	SJ	229.00±8.54	228.00±7.00		SJ	9.09±0.65^a^	9.30±0.89^a^
	SH	228.00±8.00	227.33±6.43		SH	10.16±1.06^a^	9.06±0.55^a^*
Final weight (g)	NC	403.33±7.77	403.33±7.77^c^	Kidney (g)	NC	1.51±0.26	1.51±0.26
	NB	385.00±16.85	360.67±3.21^a^		NB	1.65±0.17	1.71±0.16
	KJ	376.00±9.54	359.33±1.53^a^		KJ	1.71±0.15	1.71±0.13
	SJ	382.67±13.58	357.33±2.31^a^		SJ	1.65±0.06	1.70±0.05
	SH	386.67±7.02	374.67±6.11^b^		SH	1.71±0.06	1.65±0.14
Body weight gain (g/day)	NC	2.73±0.27	2.73±0.27^b^	Heart (g)	NC	1.00±0.18^ns^	1.00±0.18^ns^
	NB	2.48±0.29	2.12±0.17^a^*		NB	0.91±0.09	0.87±0.06
	KJ	2.44±0.21	2.10±0.08^a^*		KJ	0.94±0.06	0.91±0.10
	SJ	2.44±0.14	2.05±0.12^a^*		SJ	0.89±0.04	0.94±0.03
	SH	2.52±0.02	2.34±0.19^a^		SH	0.97±0.16	0.83±0.02
Feed intake (g/day)	NC	21.38±2.74^ns^	21.38±2.74^ns^	Total fat (g)	NC	40.83±1.43^c^	40.83±1.43^b^
	NB	23.43±1.86	22.92±2.45		NB	39.40±4.49^bc^	32.04±1.93^a^
	KJ	24.24±3.92	24.95±2.35		KJ	34.50±0.81^ab^	32.06±0.59^a^*
	SJ	23.59±0.24	22.58±0.95		SJ	33.15±1.22^a^	31.93±1.78^a^
	SH	24.19±1.40	26.37±0.63		SH	34.55±1.46^ab^	32.62±1.38^a^
FER	NC	0.13±0.03^ns^	0.13±0.03^ns^				
	NB	0.11±0.00	0.09±0.02				
	KJ	0.10±0.01	0.08±0.01				
	SJ	0.10±0.00	0.09±0.01				
	SH	0.10±0.01	0.09±0.01				

NC, AIN-93M; NB, normal brown rice; KJ, Keunnunjami; SJ, Superjami; SH, Superhongmi; FER, feed intake ratio; NS, not significantly different.

Values are means±SD (*n*=5).

a–c Means in the same column per parameter with different letters are significantly different at *P*<0.05 by Tukey's range test.

* on the germinated rice group indicates significant difference (*P*<0.05) by independent *t*-test between germinated and non-germinated samples.

### Supplementation of germinated rice improves plasma lipid profile of OVX rats

The analysis of the effects of the rice cultivars on the plasma lipid profile of the animals following ovariectomy is shown in Table 3. Plasma TC, TG, and non-HDL-cholesterol were measured, and OVX rats fed with non-germinated rice cultivars had significantly higher values relative to their germinated group counterparts. Between the non-germinated rice samples, animals fed with NB rice had significantly higher plasma cholesterol profiles than the animals fed with pigmented rice cultivars. Also, their HDL-cholesterol was lower than that of the rice-treated groups. On the other hand, all germinated rice cultivar-fed groups showed significantly lower TG and TC levels relative to the non-germinated rice-fed groups. Although the supplementation of non-germinated rice lowered the TG and TC levels compared to the normal diet, the consumption of germinated rice further normalized these lipid-level indicators. Also, the OVX rats fed with germinated rice showed significantly higher HDL-cholesterol levels. The non-HDL and HDL-cholesterol were significantly lower and higher for the rats fed with germinated rice relative to those fed non-germinated rice, respectively. The purple rice cultivars KJ (2.15±0.04 mmol/L) and SJ (2.18±0.01 mmol/L), in particular, showed the highest HDL-cholesterol level among all germinated groups. Furthermore, the HTR and AI values of OVX rats fed with germinated rice cultivar were significantly higher and lower, respectively, than the animals fed with non-germinated rice cultivars. Among all the groups, rats that were fed germinated KJ showed an overall good HTR (50.39±0.86) and AI (0.98±0.03) value relative to the other groups, which shows that this group had the least risk of developing cardiovascular diseases like dyslipidemias. On the other hand, rats fed with the non-pigmented NB showed the least desirable HTR and AI compared with the other rice-treated groups. Overall, the results showed that, among the rice cultivars included in this study, germinated purple rice grains KJ and SJ had the best lipid profile followed by the red rice cultivar SH, and then the non-pigmented NB.

**Table 3:** Plasma lipid profile analysis of OVX rats fed with non-germinated and germinated rice grains

		Non-germinated	Germinated			Non-germinated	Germinated
TC (mmol/L)	NC	6.43±0.10^c^	6.43±0.10^c^	AI	NC	2.89±0.09^d^	2.89±0.09^d^
	NB	5.46±0.14^b^	5.07±0.13^b^*		NB	2.29±0.04^c^	1.63±0.09^c^*
	KJ	4.59±0.05^a^	4.27±0.08^a^*		KJ	1.53±0.01^a^	0.98±0.03^a^*
	SJ	4.56±0.03^a^	4.34±0.14^a^*		SJ	1.52±0.04^a^	0.99±0.06^a^*
	SH	5.41±0.09^b^	5.02±0.13^b^		SH	2.09±0.03^b^	1.41±0.07^b^*
TG (mmol/L)	NC	1.24±0.06^b^	1.24±0.06^c^	FFA (mmol/L)	NC	2.05±0.09^b^	2.05±0.09^d^
	NB	1.19±0.05^ab^	0.98±0.04^b^*		NB	1.67±0.08^b^	0.70±0.05^ab^*
	KJ	1.09±0.05^a^	0.92±0.02^a^		KJ	1.28±0.10^a^	0.62±0.08^a^*
	SJ	1.19±0.04^a^	0.93±0.03^a^*		SJ	1.15±0.05^a^	0.85±0.05^bc^*
	SH	1.18±0.04^ab^	1.00±0.03^b^*		SH	1.27±0.10^a^	0.92±0.10^c^*
HDL (mmol/L)	NC	1.65±0.02^a^	1.65±0.02^a^	Phospholipids (mmol/L)	NC	0.73±0.00^a^	0.73±0.00^a^
	NB	1.66±0.02a	1.93±0.03^b^*		NB	0.98±0.01^b^	1.19±0.03^c^
	KJ	1.81±0.03^b^	2.15±0.04^d^*		KJ	1.15±0.11^c^	1.20±0.09^c^
	SJ	1.81±0.02^b^	2.18±0.01^d^*		SJ	0.98±0.05^b^	1.30±0.02^c^
	SH	1.75±0.02^b^	2.08±0.02^c^*		SH	0.83±0.01^ab^	0.95±0.02^b^
Non-HDL (mmol/L)	NC	4.78±0.11^c^	4.78±0.11^c^	GOT (Karmen/mL)	NC	49.78±4.57^c^	49.78±4.57^b^
	NB	3.80±0.11^b^	3.14±0.15^b^*		NB	28.94±1.68^b^	11.52±1.68^a^*
	KJ	2.78±0.02^a^	2.12±0.06^a^*		KJ	21.19±2.90^a^	10.55±1.68^a^*
	SJ	2.75±0.05^a^	2.15±0.13^a^*		SJ	27.00±2.90^ab^	14.42±1.68^a^*
	SH	3.66±0.07^b^	2.94±0.14^b^*		SH	25.06±1.68^ab^	15.39±2.90^a^*
HTR (%)	NC	25.71±0.61^a^	25.71±0.61^a^	GPT (Karmen/mL)	NC	43.45±1.68^c^	43.45±1.68^d^
	NB	30.39±0.36^b^	38.12±1.39^b^*		NB	39.13±5.27^b^	26.95±4.57^c^*
	KJ	39.50±0.23^d^	50.39±0.86^d^*		KJ	36.08±4.57^b^	14.77±2.64^a^*
	SJ	39.66±0.66^d^	50.38±1.52^d^*		SJ	31.51±4.57^a^	17.81±4.57^b^*
	SH	32.35±0.28^c^	41.47±1.27^c^*		SH	34.56±2.64^b^	19.33±2.64^b^*

NC, AIN-93M; NB, normal brown rice; KJ, Keunnunjami; SJ, Superjami; SH, Superhongmi; NBG, germinated normal brown rice; TG, total triglycerides; TC, total cholesterol; HDL, high-density lipoproteins cholesterol; non-HDL cholesterol=TC - HDL-cholesterol, HTR (%)=(HDL cholesterol/TC)*100; AI, atherogenic index=(TC - HDL cholesterol)/HDL; GOT, glutamic oxaloacetate transaminase; GPT, glutamic pyruvate transaminase.

Values are means±SD (*n*=5).

a–d Means in the same column per parameter with different letters are significantly different at *P*<0.05 by Tukey's range test

* on the germinated rice group indicates significant difference (*P*<0.05) by independent *t*-test between germinated and non-germinated samples.

The assessment of the liver enzymes GPT and GOT was also shown. Treatment and control groups showed liver enzyme activities within the normal range of values. However, feeding germinated rice to the OVX rats further decreased the activities of these liver enzymes, indicating a lower risk of liver damage. Meanwhile, plasma FFA and phospholipids were lower and higher, respectively, in rats fed with germinated rice than in rats fed with non-germinated rice. Based on these values, feeding germinated rice to OVX rats helped prevent the accumulation of triglycerides in the liver.

### Germinated rice enhances excretion of lipids

**Fig. 2:**
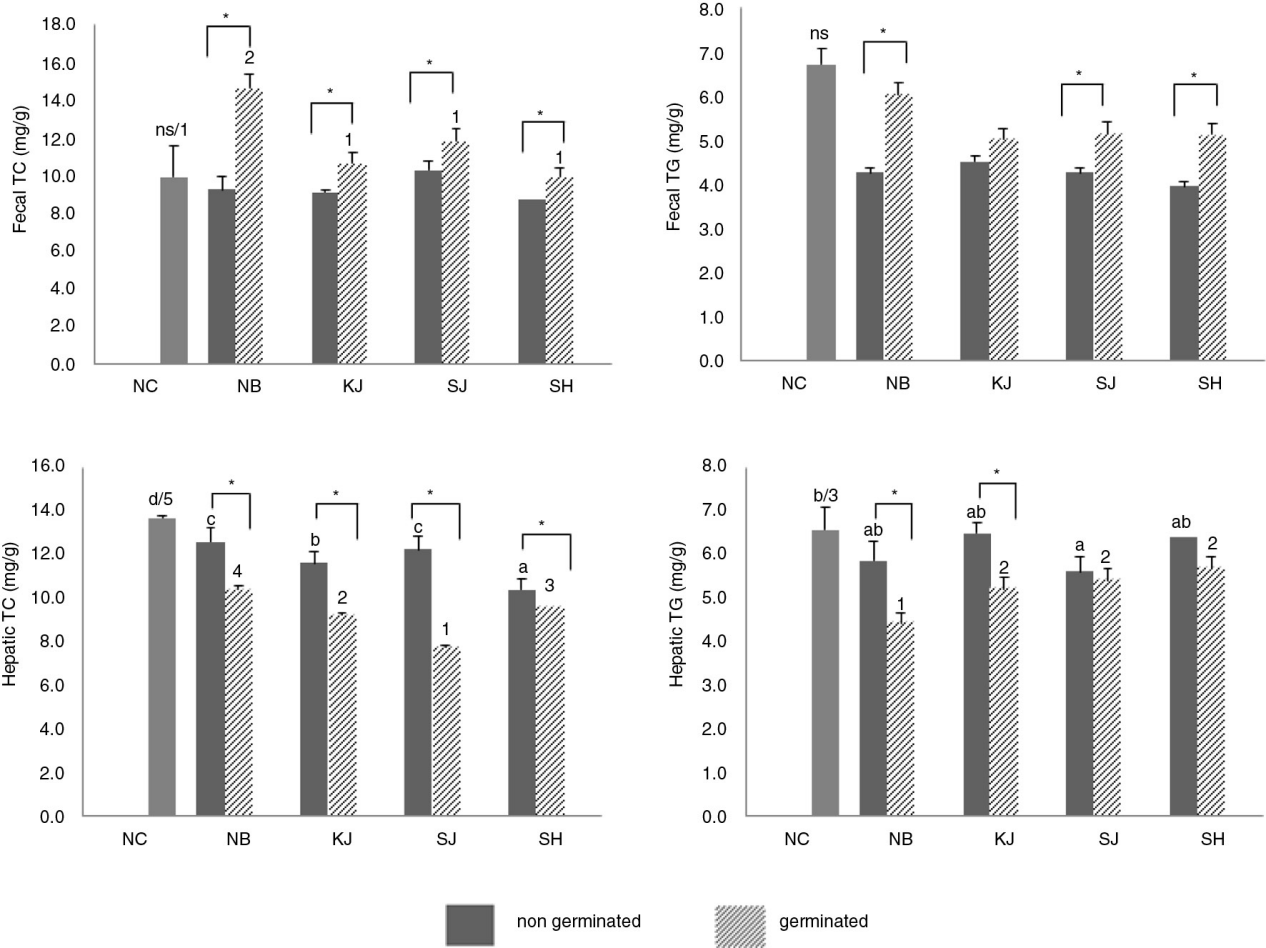
Fecal and hepatic TC and TG of OVX rats fed with non-germinated and germinated rice grains. Values are means±SD (*n=*5). ^a–d^Means in the non-germinated groups with different letters are significantly different at *P<*0.05 by Tukey's range test. ^1–5^Means in the germinated groups with different numbers are significantly different at *P<*0.05 by Tukey's range test. (*) between germinated and non-germinated samples per rice group indicates significant difference (*P<*0.05) by independent *t*-test. NC; AIN-93M, NB; normal brown rice, KJ; Keunnunjami, SJ; Superjami, SH; Superhongmi.

The significant increase in the fecal TC and TG of the OVX rats fed with germinated rice cultivars relative to non-germinated rice is shown in Fig. 2. There was no significant change in the fecal TG of both non-germinated and germinated groups. However, a significant increase in the excretion of fecal TG in non-germinated and germinated groups was observed in all rice-treated groups. Fecal TC also showed no significance in the non-germinated group, but a significant increase was observed when germinated rice cultivars were consumed by the OVX rats.

### Germinated rice decreases hepatic lipid accumulation

**Fig. 3:**
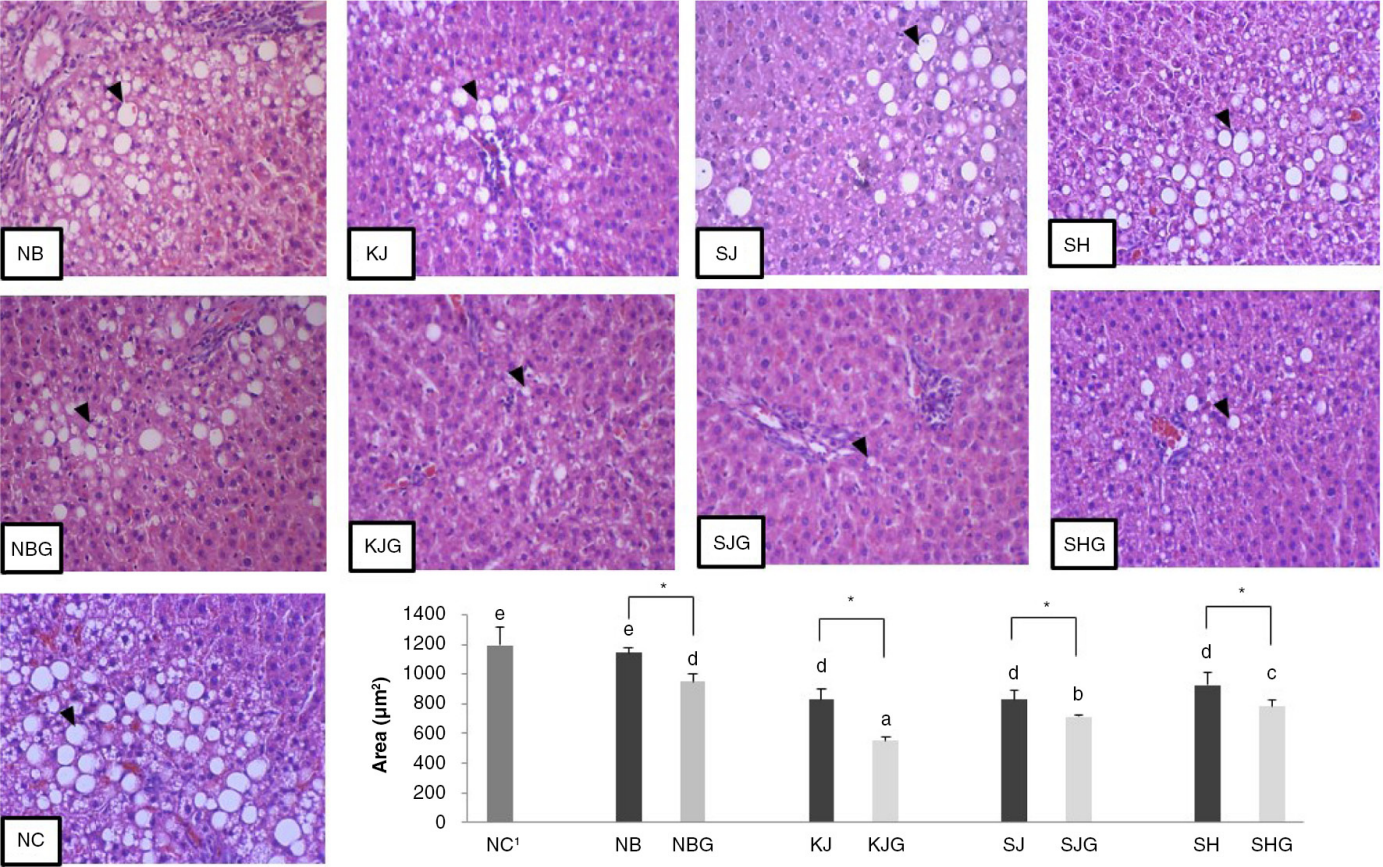
Histological images (200×) of liver sections stained with hematoxylin and eosin (H&E) and area of the stored lipids in the liver of OVX rats fed with non-germinated and germinated rice grains. Values are means±SD (*n=*5). ^a–e^Means in the same group with different superscripts are significantly different at *P<*0.05 by Tukey's range test. (*) on the non-germinated rice group indicates significant difference (*P*<0.05) by independent *t*-test between germinated and non-germinated samples. ^1^NC; AIN-93M, NB; normal brown rice, KJ; Keunnunjami, SJ; Superjami, SH; Superhongmi, NBG; germinated normal brown rice, KJG; germinated Keunnunjami, SJG; germinated Superjami, SHG; germinated Superhongmi. The black arrows indicate the presence of lipid vacuoles in the liver tissue sample.

Figure 2 also summarized the hepatic TC and TG of rats fed with non-germinated and germinated rice. Results showed that germinated rice consumption lowered the hepatic TG and TC levels as compared with their non-germinated counterpart. There was a significant decrease in the hepatic TC and TG of all animals fed with germinated rice relative to the non-germinated groups, except for the hepatic TG of the animals fed with germinated SJ and SH. Among the animals fed with germinated rice, the non-pigmented NB group had the significantly highest hepatic TC, which indicated that more lipids were being absorbed in the livers of these animals. From these findings, germinated rice cultivar consumption prevented hepatic lipid accumulation, which corresponds to the results obtained in the plasma lipid profiles of the OVX rats. On the other hand, liver tissue samples of OVX rats fed with NC diet showed severe fatty changes especially around bile canaliculi, as shown in Fig. 3. Initially, small vacuoles appeared first and then combined with each other, forming larger vacuoles. As observed, the cytoplasm of hepatic cells in rats supplemented with non-germinated rice groups was occupied by fat, and the nucleus was pushed at the side of the cell membrane. Moreover, in the non-germinated rice groups, the NB (1143.24±38.16 µm^2^) rice group had more widespread lipid vacuoles in the parenchyma cells than the pigmented rice groups following the order SH > SJ > KJ, respectively. In addition, there was no significant difference between the two purple rice cultivars, KJ and SJ, and they had lesser hepatocyte degeneration when compared with the red strain cultivar SH. Germinated groups, on the other hand, all showed fewer lipid vacuoles within the hepatocytes and, with regard to the area occupied by the lipids deposited in the liver, they had fewer than their non-germinated group counterparts. Also, Fig. 3 summarized the area occupied by the adipose cells in liver hepatocytes. Overall, the group fed germinated had a smaller area than their counterparts who were fed non-germinated rice. These hepatochemistry results coincided with the results obtained in the liver weight (Table 2) as well as the hepatic cholesterol profile (Table 3). The area occupied by more lipid cells in the liver corresponds to an increase in weight and in the amount of hepatic cholesterol retained in the liver. Furthermore, germinated purple rice KJ (550.83±25.15 µm^2^) had the smallest total area of lipids, followed by germinated SJ (712.60±14.11 µm^2^)<SH (783.32±46.14 µm^2^)<NB (947.60±55.50 µm^2^), respectively.

### Supplementation of germinated rice enhances lipolysis and inhibits lipogenesis

Lipid-regulating enzymes in the adipose tissues are summarized in Table 4. OVX rats fed with non-germinated rice showed a significant increase in the FAS, ME, and G6PD enzyme activities relative to the germinated rice-treated groups. On the contrary, rats fed with germinated rice had higher β-oxidation and CPT enzyme activities. The lipid enzyme activities had a significant difference between the non-germinated and germinated groups, except for the β-oxidation, which showed no significant difference among all treatment groups. The results revealed that, among the rice grain cultivars investigated, purple rice KJ and SJ had better lipolysis and poorer lipogenesis activities.

**Table 4:** Adipocytic lipid-regulating enzyme and β-oxidation activities of OVX rats fed with non-germinated and germinated rice grains

	Rice sample	Non-germinated	Germinated
FAS (µmol/min/mL protein)	NC	85.69±4.13^b^	85.69±4.13^c^
	NB	84.69±5.40^b^	22.98±2.16^b^
	KJ	55.46±5.40^a^	13.99±2.84^a^*
	SJ	54.46±1.56^a^	15.99±1.56^ab^*
	SH	64.45±3.43^a^	20.23±2.70^ab^*
ME (µmol NAPDH/min/mg protein)	NC	60.50±3.68^c^	60.50±3.68^d^
	NB	43.36±3.44^b^	25.21±1.85^c^*
	KJ	31.06±3.10^a^	11.70±1.85^a^*
	SJ	37.11±1.52^ab^	18.55±1.94^b^*
	SH	38.52±3.05^ab^	19.97±1.05^bc^*
CPT (µmol NAPDH/min/mg protein)	NC	49.76±1.40^a^	49.76±1.40^a^
	NB	52.94±2.66^ab^	60.62±2.97^a^*
	KJ	76.63±7.00^d^	86.21±4.36^c^*
	SJ	66.82±3.28^cd^	80.50±1.90^c^*
	SH	62.37±4.24^bc^	67.49±1.83^b^
G6PD (µmol NAPDH/min/mg protein)	NC	163.21±2.51^c^	163.21±2.51^d^
	NB	161.21±4.27^c^	43.63±1.15^c^*
	KJ	131.57±6.11^ab^	26.31±1.53^a^*
	SJ	119.58±7.02^a^	30.64±3.21^ab^*
	SH	136.56±6.02^b^	36.31±1.53^b^*
β-oxidation (µmol NAPDH/min/mg protein)	NC	1.31±0.43	1.31±0.43
	NB	1.42±0.39	2.29±0.54
	KJ	1.99±0.41	2.32±0.28
	SJ	1.89±0.28	2.19±0.39
	SH	1.83±0.21	2.11±0.33

NC, AIN-93M; NB, normal brown rice; KJ, Keunnunjami; SJ, Superjami; SH, Superhongmi; NBG, germinated normal brown rice; FAS, fatty acid synthase; ME, malic acid enzyme test; CPT, carnitoyl transferase; G6PD, glucose-6-phosphate dehydrogenase.

Values are means±SD (*n*=5).

a–d Means in the same column per parameter with different letters are significantly different at *P*<0.05 by Tukey's range test.

* on the germinated rice group indicates significant difference (*P*<0.05) by independent *t*-test between germinated and non-germinated samples.

## Discussion

Rice is one of the most widely consumed cereal foods by many populations. In recent years, many researches have demonstrated the functional effects of rice, and continuous research has been conducted to further develop more nutritious and functional rice varieties. Also, many research efforts have been devoted to newly develop rice cultivars, especially those that are pigmented, for they are said to contain high levels of anthocyanins and are therefore have a lot of health benefits. Contributing to these health benefits are various bioactive compounds such as flavones, phenolic compounds, tannins, GABA, tocopherols, tocotrienols, γ-oryzanol, and ferulic acids. Some of these bioactives are said to have hypolipidemic effects (22).

In line with this, the study showed the differences in the quantities of some of these bioactive compounds that have hypolipidemic effects. Studies have illustrated that the higher the γ-oryzanol, phytosterols, and fibers present in the rice grain, the better the hypolipidemic effects and the ability to inhibit the enzyme responsible for cholesterol synthesis (23). Also, an increase in the γ-oryzanol content brought about by germination further lowers the lipid profile of the OVX rats by inhibiting cholesterol absorption in the intestines, and thus also increasing fecal cholesterol excretion (22). Ferulic acid, on the other hand, is said to terminate the free radical reactions, thereby reducing the risk for cardiovascular diseases, and is a good anti-oxidant that facilitates the uptake and degradation of cholesterol in the liver (24). Furthermore, an increase in the levels of these bioactives after germination may be partly responsible for the decrease in the total fat of the rat groups. Also, it shows the effectiveness of germinated rice in lowering adipocyte hypertrophy and hyperplasia, despite the higher risk brought about by the decrease in estrogen hormone levels of these OVX rats (25). In addition to γ-oryzanol and ferulic acids, this study also investigated the tannin content, which is believed to contribute to the synergistic hypolipidemic effects of the bioactives found in rice. Tannins, one of the phytochemicals found in rice, promoted the excretion of fecal cholesterols, which leads to a decreased absorption of dietary cholesterol as well as lower plasma and hepatic cholesterol, as shown by the results obtained from the OVX rats feeding with germinated rice relative to non-germinated rice (26).

Meanwhile, Hongu et al. (7) elucidated that high circulating concentrations of TC, LDL-cholesterol, and TG, combined with low levels of HDL-cholesterol, are established biomarkers of risk for cardiovascular diseases and dyslipidemias. In normal lipid metabolism, the dietary lipids are absorbed in the body and package into very low-density lipoproteins (VLDL). By the action of the enzyme lipoprotein lipase, these VLDLs will be converted to FFA, which are used by peripheral tissues as an energy source (27). However, if lipid intake is higher and supply exceeds the demand, these lipids will be stored as fat in the adipose tissues for future use. Finally, if these unused fats remain as stored fats, the fatty acids in the plasma will exceed threshold, thus causing dyslipidemias and other cardiovascular diseases (28). On the other hand, non-HDL-cholesterol levels should be kept lower to prevent cardiovascular diseases, whereas HDL-cholesterol, regarded as ‘good’ cholesterol, should have a higher value to be more beneficial (2). HDL is important because it carries the cholesterol and cholesterol esters from the peripheral cells or tissues to the liver, which is a very important step in the maintenance of normal lipid metabolism (29). The better lipid profile among the pigmented rice varieties is attributed to the presence of high levels of bioactives, which are enhanced by germination, and include anthocyanins, phenolic compounds, γ-oryzanol, and ferulic acids. The bioactives neutralize the free radicals in the body and thus are responsible for their lipid-lowering properties (6). The neutralization of free radicals also prevents fat accumulation, such that the amount of dietary lipids is just enough for the energy source. Among the rice cultivars studied, purple rice varieties (KJ and SJ) have, in general, a better lipid profile. This may be due to the limited number of pigments found in the reddish cultivar SH relative to purple varieties that are sources of these bioactive compounds as elucidated from previous investigations (29).

Furthermore, germination is said to enhance all the rice minerals and nutrients such as tocopherols, γ-oryzanols, ferulic acids as well as the dietary fibers present by activating the dormant enzymes present in the rice grain (10). The consumption of foods high in dietary fiber, such as germinated rice, is said to disrupt nutrient absorption, which results in increased fecal excretion of energy, proteins, and even fats, and a rapid cholesterol turnover (30). Consequently, the fecal lipid values will then increase as a result of decreased intestinal absorption, as revealed in the study results. This also correlates with the γ-oryzanol values result in which the higher the γ-oryzanol of a rice grain, the more the rice can inhibit cholesterol absorption, and thus increase fecal excretion of bile acids (31).

The liver is the major site for cholesterol biosynthesis; therefore, the hepatic TC and TG levels are good indicators of dyslipidemias. When the values of these parameters are increased, the risk of atherosclerotic process also increases progressively (32). The process by which the HMG-CoA is converted to mevalonate via the microsomal enzyme HMG-CoA reductase occurs in the liver. When the process of conversion is enhanced, the TC level is also increased. Therefore, the inhibition of this enzyme is the first rate-limiting step for cholesterol synthesis (33). Furthermore, liver steatosis is a clinical condition wherein triglyceride fat vacuoles accumulate in the liver cells. The liver fat content reflects the equilibrium between FFA flux through lipolysis, fatty acid oxidation, de novo lipogenesis, and VLDL secretion into the bloodstream. In fact, abnormal high levels of FFA are associated with metabolic diseases (34). Hübscher (35) stated that if there is an increase in the delivery of FFA to the liver coupled with impaired fatty acid metabolism in hepatocytes, there will be a net accumulation of triglycerides in the liver. This is also correlated with the occurrence of metabolic diseases such as dyslipidemias that occur when the rate of FFA entering the liver is greater than the output rate (36). However, the data show that germinated rice inhibits triglycerides accumulation in the liver cells, thus lowering the risk of dyslipidemia occurrence on those who consumed these rice varieties.

Last, the lipid-regulating enzymes are important in fatty acids and cholesterol biosyntheses. A decrease in the activities of these lipogenic enzymes results in the unavailability of the fatty acids needed for triglyceride production (37). The enzyme CPT-1 is the main regulatory enzyme in long-chain fatty acid oxidation for it catalyzes the conversion of fatty acid-CoA into fatty acid–carnitine before entering the mitochondrial matrix (38). Together with β-oxidation, they constitute the lipolytic process. Meanwhile, FAS, G6PD, and ME enzymes are involved in the fatty acid biosynthesis or lipogenesis, for they all provide the NAPDH needed for the synthesis of fatty acids in the pentose phosphate pathway (39). Thus, normal lipid regulation and metabolism are all maintained by these enzymes. OVX rat groups fed with germinated rice, relative to non-germinated rice groups, have significantly lower FAS, ME, and G6PD enzyme activities and higher CPT and β-oxidation enzyme activities. These results indicate that the process of germination, in addition to higher phytochemical contents of the pigmented rice cultivars, enhanced the fatty acid oxidation process, which in turn reduces triglyceride accumulation (40). This reduction in lipogenesis brought about by consuming germinated rice could be responsible for the marked reduction in the TC, TG, and fatty acid levels in the plasma as well as the higher cholesterol excretion in the feces.

## Conclusions

This study demonstrated the effects of the newly bred Korean non-germinated and germinated pigmented rice cultivars on OVX rats in comparison with the non-pigmented normal brown rice. All rat groups were fed with 20% rice per total diet and showed that the supplementation of germinated rice for some groups, particularly pigmented rice cultivars, resulted in better a lipid profile compared to the groups that consumed non-germinated rice cultivars. In addition, germination increased the quantities of the bioactive compounds that are responsible for the hypolipidemic activities of these rice grains. Overall, the results obtained showed the potential for using germinated rice, blackish-purple cultivars KJ and SJ in particular, as functional foods with enhanced benefits for the prevention and occurrence of dyslipidemias.
